# Smaller Copper Oxide Nanoparticles have More Biological Effects Versus Breast Cancer and Nosocomial Infections Bacteria

**DOI:** 10.31557/APJCP.2021.22.3.893

**Published:** 2021-03

**Authors:** Ardeshir Abbasi, Khodayar Ghorban, Farshad Nojoomi, Maryam Dadmanesh

**Affiliations:** 1 *Infectious Diseases Research Center, Aja University of Medical Sciences, Tehran, Iran. *; 2 *Department of Immunology, School of Medicine, Aja University of Medical Sciences, Tehran, Iran. *; 3 *Department of Microbiology, School of Medicine, Aja University of Medical Sciences, Tehran, Iran. *; 4 *Department of Infectious Diseases, School of Medicine, Aja University of Medical Sciences, Tehran, Iran. *

**Keywords:** Copper oxide nanoparticle, acinetobacter baumannii, Staphylococcus epidermidis, nosocomial infections

## Abstract

**Background and Objectives:**

Despite promising successes in developing new drugs and pharmaceutical biotechnology, infectious diseases and cancer are still the principal causes of mortality and morbidity globally. Therefore, finding effective ways to deal with these pathogens and cancers is critical. Metal nanoparticles are one of the new strategies to combat bacteria and cancers.

**Methods:**

We examined the antimicrobial activity of 30 and 60 nm copper oxide nanoparticles (CuO-NPs) against Acinetobacter baumannii and Staphylococcus epidermidis bacteria responsible for nosocomial infections in standard and clinical strains and anti-cancer activity against 4T1 cell line as malignancy breast cancer cells. Synthesis of CuO-NPs was performed by a one-step reduction method and confirmed by DLS and TEM microscopy at 30 and 60 nm sizes. The antibacterial and anti-cancer activities of the nanoparticles were then investigated against the aforementioned bacteria and breast cancer.

**Results:**

Using disk, well, MIC, MBC methods, and viability/bacterial growth assay, 30 nm CuO NPs were found to have more antibacterial activity on standard and clinical strains than 60 nm CuO NPs. On the other hand, using MTT, apoptosis, and gene expression method, 30 nm nanoparticles were found to have more anti-cancer potential than 60 nm CuO NPs.

**Conclusions:**

Our findings implicate CuO-NPs to possess antimicrobial and anti-cancer effects and more significant potential in smaller sizes, suggesting their pharmaceutical and biomedical capacity.

## Introduction

Nosocomial infections (NIs) are among the most critical problems affecting all hospitals worldwide and one of the health issues among all societies. Controlling the spread of these infections remains a significant challenge, especially in hospitals. Moreover, cancer is one of the biggest medical concerns. Breast cancer is the second leading cause of death among women all around the world. NIs and cancer lead to increased morbidity, mortality, cost, and hospitalization, as well as inflicting staff and economic pressure to the health centers (Motbainor et al., 2020). The rapid emergence of antibiotic-resistant bacteria has affected the efficacy of infectious disease treatment and the health of millions of patients worldwide (Kajihara et al., 2020). Multi-drug resistance (MDR) has been recently reported to be responsible for 700,000 annual deaths and is predicted to reach 10 million deaths in 2050, providing that new antibacterial agents are not being developed (Maddila et al., 2020). The two bacteria responsible for the majority of nosocomial infections are *Staphylococcus epidermidis* (*S. epidermidis*) and *Acinetobacter baumannii *(*A. baumannii* (*Staphylococci *are critical pathogens for humans, which cause a wide range of dangerous systemic diseases (skin infections and opportunistic infections) (Da et al., 2017). Staphylococcus epidermidis (S. epidermidis) (coagulase-negative, gram-positive cocci) is one of the most important opportunistic pathogens in medical devices; therefore, it is mainly associated with medical-device infections. S. epidermidis is one of the common-colonized bacteria in human skin (Amaro et al., 2019). *Acinetobacter baumannii* (*A. baumannii*) is an aerobic and polymorphic gram-negative bacterium, which is highly resistant to common antibiotics because it is isolated from the hospital gut (Grandesso et al., 2014; Atik et al., 2018). Therefore, these bacteria can be considered to impose future threats and cause hospital infections. Importantly, instant actions to deal with these pathogens is one of the global health priorities. Breast cancer is still important similar to 2019, that was reported with an outbreak of 268,600 new cases in the United States. 4T1 breast carcinoma is a triple-negative tumor cell line that is highly invasive, metastatic, and tumorigenic (Abbaset al., 2020; Abbasi et al., 2021). Furthermore, the number of cancer patients is increasing every day. Therefore, finding effective ways to tackle these pathogens and cancers is critical. 

**Figure 1:**
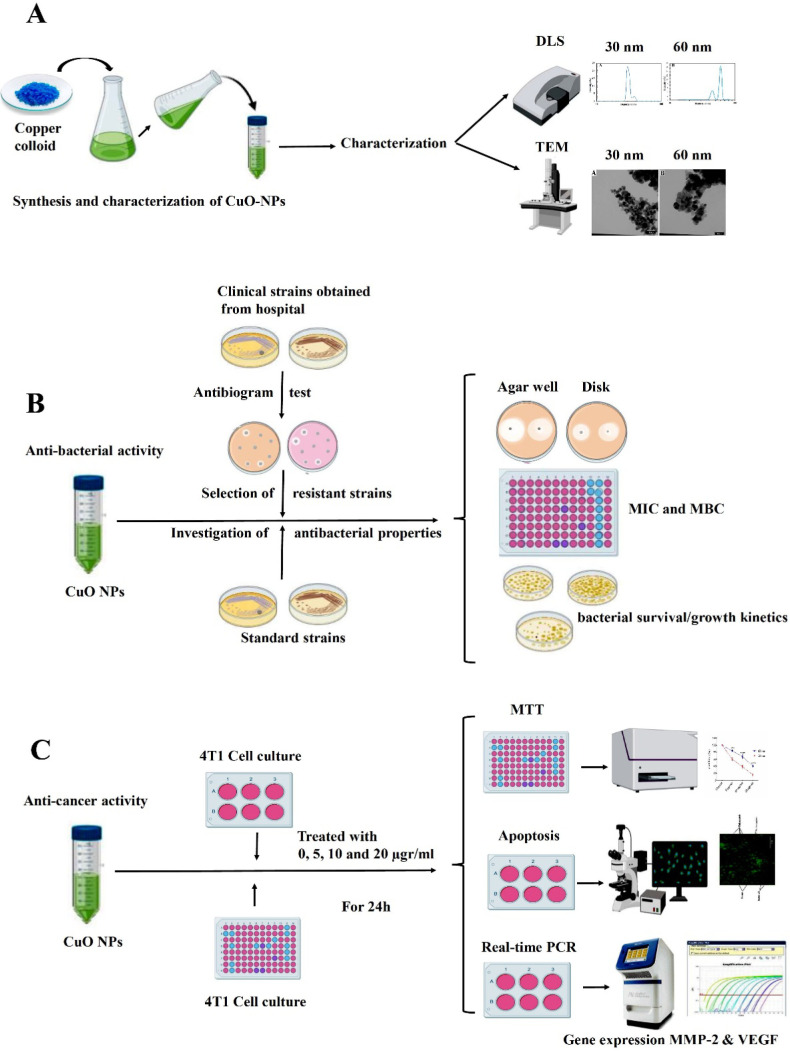
Schematic form of Project Design. A, In the first step, copper oxide nanoparticles are synthesized using the one-step reduction method, then size and morphology characterization of CuO-NPs was performed by DLS and TEM methods. B, In the second step, bacteria resistant to common antibiotics were isolated from the infectious section of the hospital. On the other hand, Standard bacterial strains were prepared. Afterthought antibacterial properties of CuO-NPs on standard and clinical strains of the bacteria of A. baumannii and S. epidermidis were investigated by disk, well diffusion testing, MIC, MBC, and bacterial survival/growth kinetics methods. C, In the third step, the anti-cancer activity of CuO NPs on the 4T1 breast cancer cell line was evaluated by MTT, Apoptosis, and Real-time PCR for gene expression of MMP-2 and VEGF as metastatic and angiogenesis of cancer cell

Nanoscale materials have long been the focus of researchers and materials scientists. The reduction in particle size from micro to nano has led to the emergence of specific properties such as hardness, high electrical conductivity, increased surface-to-volume ratio, high reactivity, and altered biological activity (Ilinskaya et al., 2019). Such properties enable the particles to interact with microbes, bacterial membranes, and cancerous cells more meritoriously. Hence, they gradually release metal ions along the membrane (Yang et al., 2012). The toxic activity of metal NPs, including gold, copper, zinc, and titanium, has been of great interest for treatment options (Babaei et al., 2017; Kukia et al., 2018; Kukia et al., 2018). More attention has been focused on silver and copper nanoparticles that have intrinsic antibacterial, anti-cancer, and antioxidant properties because of their comfortable and rapid use, availability, low-concentration toxicity, and lack of environmental hazards (Ali et al., 2020). CuO-NPs, with different morphological structures, may have multifunction biology activities such as antibacterial and anti-cancer roles, which are produced using various synthetic pathways such as precipitation (Mayekar et al., 2014; Kukia et al., 2018), electrochemical synthesis (Katwal et al., 2015), thermal decomposition (Shahsavani et al., 2016), sono-chemical synthesis (Silva et al., 2019), heat treatment approach (Baqer et al., 2018), nonionic water-in-oil micro-emulsions (Dodoo-Arhin et al., 2012), sol-gel synthesis (Dörner et al., 2019) and Laser Ablation in Open Air (Fernández-Arias et al., 2020). Previous studies have also shown that the effect of nanoparticles is size-dependent (Albanese et al., 2012). Nevertheless, studies aimed at assessing the professional performance of NPs, adverse effects, biosecurity, and toxicology are still scarce. Therefore, in this study, a one-step reduction method was used to prepare an aqueous colloidal aqueous solution without toxicants to produce a strong disinfectant at multi-biological effects. Moreover, we compared the biological effect of CuO-NPs at two different sizes of 30 and 60 nm on two *A. baumannii* and *S. epidermidis *bacteria, representing gram-negative and positive bacteria respectively, responsible for nosocomial infections in standard and clinical strains and 4T1 breast cancer cell line as representative of malignant cells to an evaluation of their pharmaceutical and biomedical properties (see Figure 1).

## Materials and Methods

*Materials and strains of the bacteria investigated*

This fundamental and applied study was accompanied under the supervision of the Ethics Committee of Army Medical University. In this study, standard strains were prepared of *A. baumannii* (ATCC BAA-747), and *S. epidermidis* (ATCC 49461) from Iranian Biological Research Center, while clinical strains were obtained from Army Hospital. They were selected as representative of the Gram-positive (*S. epidermidis*) and negative (*A. baumannii*) bacteria responsible for antibiotic resistance and hospital infections. Culture media, nutrient broth, Mueller Hinton Agar, BHI, Peptone Water, brain agar, and Tryptic Soy Broth were used. All culture media were purchased from Merck Company (Merck-Germany).

*Investigation antibiotic-resistant clinical strains of A. baumannii and S. epidermidis bacteria by antibiogram test*

To performed this test, each clinical bacteria strain was cultured distinctly using a germ-free swab on Mueller Hinton agar. Antibiotic discs, such as Gentamicin, Cefpodoxime, Ciprofloxacin, Cefdinir, Trimethoprim-sulfamethoxazole, Novobiocin, and Erythromycin, were used against *S. epidermidis* and Piperacillin, Imipenem, Gentamicin, Ciprofloxacin, Cefotaxime, and Ampicillin-sulbactam were used against A. baumannii. Then the cultured plates had been incubated 24 hours at 37°C. After incubation, the diameter of the inhibition-zone shaped around the disks was measured using a caliper and determined in the mm unit.

*Synthesis and characterization of CuO-NPs*

A one-step reduction method was used to prepare an aqueous copper colloid solution, described by Han. et al, (2011) (Han, Song et al. 2010). For this purpose, firstly, 0.25 gr of copper sulfate (CuSO_4_.5H_2_O) was dissolved in 100 ml distilled water. After that, 5 gr of Polyvinylpyrrolidone (PVP-K30) (Merck-Germany) was dissolved in the equipped aqueous solution. Then, 0.25 gr of NaBH_4_ was used to reduce the copper oxide under stirring situations and at the controlled temperature (25 ºC) and atmosphere (1 atm). Accordingly, the color of the copper sulfate solution altered to green, and the color of the solution turned in brown after a couple of minutes. After incubating the mixture for 30 minutes, ascorbic acid, at the obligatory amount, was added to the mixture and incubated at 60°C for 30 minutes. Finally, after completing the copper oxide reduction course, the solution was cleaned with ethanol and centrifuged at 450g to collect the copper colloid. Changed values of ascorbic acid were used to control the size of CuO-NPs. Zeta Sizer maneuver (Malvern zeta sizer nano-25) and Transmission electron microscope (TEM) (MIRA3- TESCAN) were used for evaluation of the size and morphology of CuO-NPs.

*Antimicrobial susceptibility tests of CuO-NPs*

*Disk diffusion test*

To implement this test, clinical strains and standard strains of the bacteria of *A. baumannii* and *S. epidermidis* were cultured in the BHI medium and incubated at 37°C for 24 hours. Then, the turbidity of the cultured bacterial strains was controlled by using the 0.5 McFarland standard method (Bahar Afshan-Iran) and using a spectrophotometer (Jenway 6305) at a 600 nm wavelength to achieve 1.5×10^8^ CFU/ml from each bacterial strain. Thereafter, bacteria strain was cultured distinctly using a germ-free swab on Mueller Hinton agar. After that, 20 μl of 1 mg/ml synthesized nanoparticles (30 or 60 nm) were loaded on the 6 mm sterile standard disks (Padtan-Iran) in 8 cm plates. Unloaded disks (Padtan-Iran) were also used as control. Finally, they were incubated for 24 hours at 37 °C, and then the diameter of the inhibition zone designed around the disks loaded with the nanoparticles was measured using a caliper and informed as to the mm unit.

*Agar well diffusion test*

Briefly, 0.5 McFarland standard was obtained from bacteria. After that, the bacterial strains were cultured on a regular medium, and a well was generated by using a disinfected punch inside the plate. Next, 50 μl of 1 mg/ml of CuO-NPs was injected in each well on separate plates, while the wells containing Dimethyl Sulfoxide (DMSO) (Merck-Germany) were organized as a control. Plates were incubated for 24 hours at 37°C. Then, the diameter of the inhibition region formed around the wells containing the nanoparticle was measured, expending a caliper and informed in mm unit.

*Minimal inhibitory concentration (MIC) and minimum bactericidal concentration (MBC) tests*

The protocol presented by Raeisi and colleagues (Raeisi, Tajik et al. 2016) was used to analyze the minimal inhibitory concentration (MIC) and minimum bactericidal concentration (MBC). Briefly, different concentrations of CuO-NPs were prepared by using the Tryptic Soy Broth medium. At the same time, 0.5 McFarland standard was obtained from bacteria. After that, dilutions ranging from 2 to 7 times more than the dilution of the leading solutions of CuO-NPs were equipped in tubes comprising BHI (78 to 10000 µg/ml) to determine MIC, and MBC. First, 20 μl of prepared concentrations of CuO-NPs, 160 μl BHI, and 20 μl of bacterial inoculation were added for each bacterial strain distinctly in micro-plate wells. For positive control, 200 μl of BHI medium and nanoparticle, and for the negative control, 180 μl of BHI medium along with 20 μl of bacterial inoculation were added to the wells. The micro-plates were shaken for 1 minute on the plate thermo-shaker device and incubated at 37 °C for 37 hours. After a period of time, the wells were observed in terms of creation- or non-creation of turbidity, and the first translucent well was measured to be MIC, and the second one was measured to be MBC well.

*Investigating the bacterial survival/growth kinetics*

In summary, 8 ml of BHI broth medium was initially added in four tubes, and 1 ml of 18-hour culture on the bacterial strain was inoculated into the BHI medium. Besides, 1 ml of CuO-NPs with dilutions of 0.1, 0.01, and 0.001 mg/ml was initially transferred into three tubes individually. Physiology serum had been added to the fourth tube as the control. Subsequently, media have been incubated at 37°C, and each of the considered concentrations was initially diluted at intervals of 0. 15, 30, 45, and 60 min and cultured on plates including the BHI agar channel. Subsequently, the cultured plates had been incubated at 37°C for 24 hours. After incubation, the amount of survived bacteria was calculated using the colony counter, plus the bacterium survival/growth moment curve seemed to be plotted. This evaluation was executed in three replications for each bacterial strain independently, and standard deviations of principles were calculated (p price <0.05).

*Anti-cancer potential tests of CuO-NPs*

*Cell culture and treatment*

Mouse-breast cancer cell line, namely 4T1, was purchased from the National Cell Bank of Iran (NCBI, Tehran, Iran) and was cultured in a 25-cm^2^ flask at the density of 5×10^4^ cells/cm^2^ in a regular medium (RPMI 1960 supplement with 10% FBS, 100 IU/ml streptomycin, and 1% glutamine plus 100 IU/ml penicillin) incubated in 5% CO_2_ at 37°C. After reaching confluency of 80%, cells were trypsinized, harvested, and seeded in six or 96-well plats and incubated for 24 hrs. Afterward, cells were treated with CuO NPs (30 and 60 nm) at the concentrations of 0 (as a control), 5, 10, and 20 μg/ml for 24 hrs. 

*Measurement of cell viability/proliferation by MTT assay*

MTT assay was performed to evaluated cell viability/proliferation of 4T1 cells after exposure to CuO NPs. In 96-well plates, 4T1 cancer cells (5× 10^4^ cells/well) were seeded in 200 µl regular medium overnight. After that, they were treated with different concentrations (described above) for 24hrs; the procedure previously described by Rahmani-Kukia et al. (Abbasi, Kukia et al. 2018, Rahmani-Kukia, Abbasi et al. 2020) was performed. Briefly, 20 µl of MTT solution (5 mg/ml in PBS) was added to each well and incubated for four hours at a cell culture incubator. The supernatant was discarded, and 100 µl of DMSO was added to each well. In the following, optical density (OD) was measured by an ELISA microplate reader at 540 nm. Finally, the difference in viability/proliferation between control and the treated cells was evaluated with the following equation: 

Viability rate (%): (OD of the treated cells/ OD of the control cells) × 100.

*Measurement of cell apoptosis using acridine orange(AO) and propidium iodide(PI)*

Apoptosis was determined by AO and PI. Cancer cells were seeded in six-well plates (1× 10^6^ cells/well) and were allowed to carpet the wells for 24h. After being treated with different concentrations (as described above) for 24 hrs, the supernatant was removed, and the cells were washed twice with PBS. After staining with 10 µl of AO (AO, Sigma Aldrich 10 μg/ml), cells were incubated for 15 minutes at 25°C in dark condition. 10 µl of PI (PI, Sigma Aldrich 10 μg/ml) was immediately added before measurement by a fluorescent microscope. Then the percentage of cells exhibiting apoptosis patterns was counted and calculated.

*Measurement of cell migration and angiogenesis-related gene expression*

Cancer cells were seeded in six-well plates (1× 10^6 ^cells/well) and were allowed to carpet the wells for 24 h. After treated with different concentrations (as described above) for 24 hrs, total RNA was extracted from cells using an isolation RNA kit (Thermo Fisher Scientific), according to the manufacturer’s instructions with an RNA quality of 1.9. Complementary DNA (cDNA) was synthesized using a cDNA Synthesis Kit (Thermo Fisher Scientific), based on the manufacturer’s instructions. The expression level of MMP-2 (as cell migration biomarker) and VEGF (as angiogenesis biomarker) were evaluated by Step-one plus Real-time PCR system (Applied Bio-Systems). This process was steed with primers including MMP-2 (F– GGACAAGAACCAGATCACATAC, R–CGTCGCTCCATACTTTTAAGG), VEGF (F–GGATCAAACCTCACCAAAGC, R – GCAGGAACATTTACACGTCTG) and an endogenous control gene HPRT (F – CAGGACTGAAAGACTTGCTC, R– AGGTCAGCAAAGAACTTATAGC). According to application, an analysis of fold change expression was determined. 

*Statistical analysis*

Statistical significance was performed by two-way ANOVA analysis of variance using Graph Pad Prism 7.0 for Windows (Graph Pad Software, Inc., San Diego, CA, USA). * p<0.05, ** p<0.01, *** p<0.001 and **** p<0.0001 were considered as significance level for all analyses performed. Data were presented as mean ± SD in triplicates experiments.

## Results

*Size and morphology of CuO-NPs*

**Figure 2:**
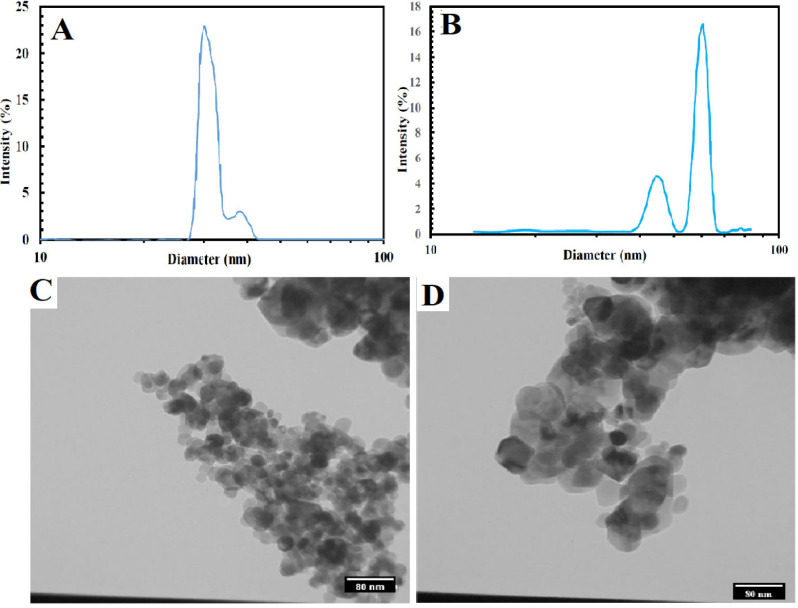
Hydrodynamic Diameter (Determining the Size) of CuO-NPs via DLS and TEM Results of Synthesized CuO-NPs. A, 30 nm of CuO-NPs. B, 60 nm of CuO-NPs. C, Morphology of CuO-NPs with 30 nm. D: Morphology of CuO-NPs with 60 nm

Investigation of the size of synthesized CuO-NPs using the Zeta Sizer device and a specified value of ascorbic acid showed that CuO-NPs were synthesized with the approximate sizes of 30 and 60 nm Transmission Electron Microscope (TEM) investigations also proved the 30 and 60 nm of CuO-NPs (see Figure 2).

*Antibiogram Test Results*

**Figure 3:**
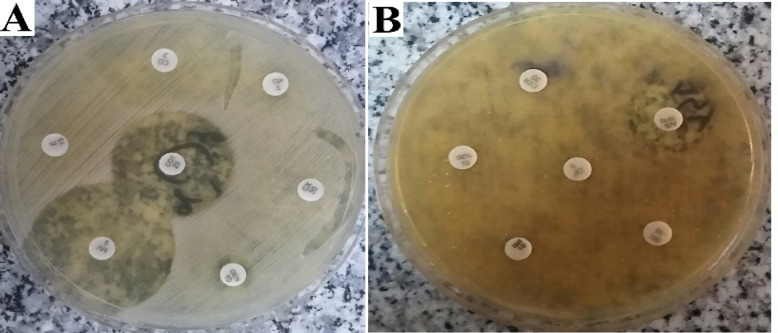
Antibiotic Resistance Clinical Strain of *S. epidermidis* and *A. baumannii *Bacteria by Antibiogram Test. A, Antibiotic disks were used for *S. Epidermidis* bacteria. B, Antibiotic disks were used for *A. Baumannii* bacteria

The results showed that *S. epidermidis *bacteria were sensitive to Trimethoprim-sulfamethoxazole and Novobiocin while resistant to other antibiogram discs (Figure-3). *A. baumannii *was only sensitive to the Ampicillin-sulbactam antibiotic disc and was resistant to other antibiotic discs (Figure 3). As a result, the clinical bacteria used in this study had antibiotic resistance.

*Results of CuO NPs antibacterial properties*

*Well diffusion and Disk diffusion test results*

As results are shown in Table 1, the mean diameter of inhibition zone resulting from the treatment of S. epidermidis standard and clinical strains were 19 ± 1.3,15 ±1.5 mm for 30 nm and 14 ± 2, 10 ± 2 mm for 60 nm CuO-NPs, respectively, whereas *A. baumannii *standard and clinical strains had 13 ± 1 and 9 ± 1.5 mm for 30 nm and 9 ± 2.5 and 5 ± 2 mm for 60 nm CuO-NPs, respectively. On the other hand, these values in the well diffusion test with 30 nm and 60 nm CuO-NPs exposed with *S. epidermidis* clinical and standard strains were 28 ± 1.5 and 26 ± 2 mm for 30 nm and 23 ± 2 and 21 ± 1 mm for 60 nm, respectively. Moreover, for A. baumannii clinical and standard strains were 18 ± 1.5, 11 ± 2 mm for 30 nm, and 14 ± 1.5 and 9 ± 1.5 mm for 60 nm, respectively. 

*MIC and MBC test results*

In brief, the data obtained in determining the MIC and MBC show that CuO-NPs had significant antibacterial effects on the standard strain compared to clinical strain on both *A. baumannii* and *S. epidermidis* bacteria. Furthermore, the smaller size (30 nm) of the CuO-NPs has more antibacterial properties than the larger size (60 nm). Table-2 shows these differences.

*Bacterial survival/growth kinetics test results*

**Figure 4:**
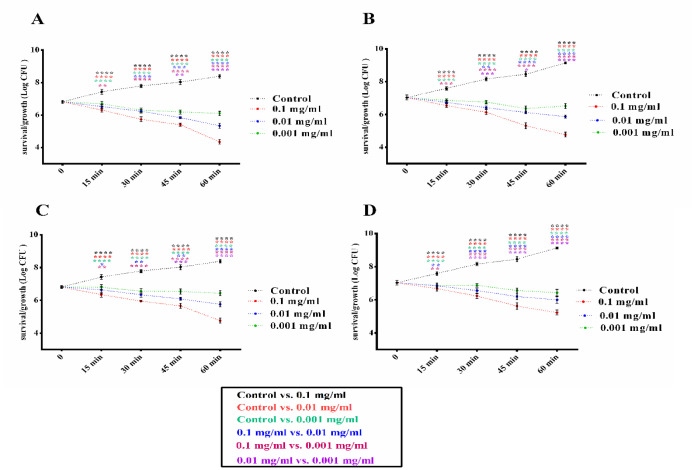
*S. epidermidis* Standard and Clinical Strains Survival/Growth Curves in the Presence of Different Concentrations (0.1, 0.01, 0.001 mg) and Different Sizes (30 or 60 nm) of CuO NPs (mg/mL). A, Standard strain treated with 30 nm size of CuO NP. B, Clinical strain treated with 30 nm size of CuO NP. C, Standard strain treated with 60 nm size of CuO NP. D, clinical strain treated with 60 nm size of CuO NP. Data shown are means ± SD (n=3) (*p < 0.05, **p < 0.01, ***p < 0.001 and ****p < 0.0001 vs similar concentrations in different conditions and each index is represented with assigned color in legend

**Figure 5:**
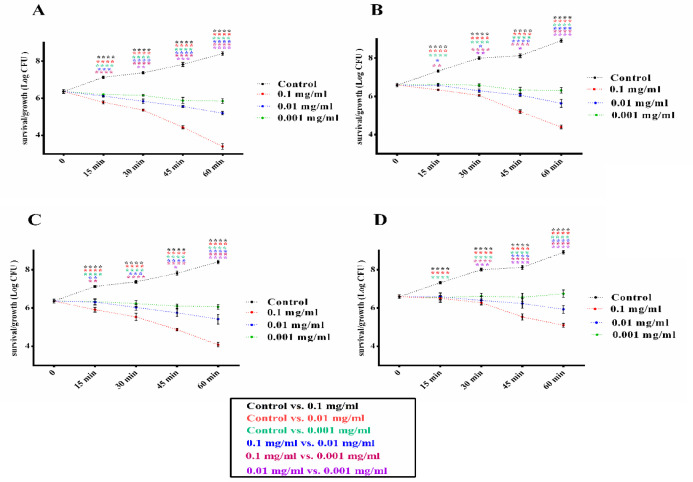
*A. baumannii* Standard and Clinical Strains Survival/Growth Curves in the Presence of Different Concentrations (0.1, 0.01, 0.001 mg) and Different Sizes (30 or 60 nm) of CuO NPs (mg/mL). A, Standard strain treated with 30 nm size of CuO NP. B, Clinical strain treated with 30 nm size of CuO NP. C, Standard strain treated with 60 nm size of CuO NP. D, clinical strain treated with 60 nm size of CuO NP. Data shown are means ± SD (n=3) (*p < 0.05, **p < 0.01, ***p < 0.001 and ****p < 0.0001 vs similar concentrations in different conditions and each index is represented with assigned color in legend

Investigation of CuO-NPs exposure in two different sizes of CuO-NPs, 30 and 60 nm, in different times, were done on A. baumannii and S. epidermidis bacteria in standard and clinical strains. In Figure 4, the results showed that CuO-NPs significantly reduced the viability and growth of *S. epidermidis *bacteria in both standard and clinical strains in both doses and at different times. Nevertheless, this decrease in viability was more significant at concentrations of 0.1 mg than 0.01 and 0.001 mg. Similarly, in Figure 5, the results showed that CuO-NPs in the dose and time-dependent manner reduced the growth and viability of *A. baumannii* in standard and clinical strains. It also had higher antibacterial activity than *A. baumannii* at a concentration of 0.1 mg and 30 nm. Interestingly, clinical strains were more resistant than standard strains of both CuO-NPs, and the 30 nm CuO-NPs were significantly more effective than the 60 nm. On the other hand, it was found that *S. epidermidis* bacteria were more sensitive than *A. baumannii* to CuO-NPs in both standard and clinical strains.

*Results of the anti-cancer potential of CuO-NPs*

*Size and concentration-dependent of CuO NPs on cancer cell viability/proliferation*

**Figure 6:**
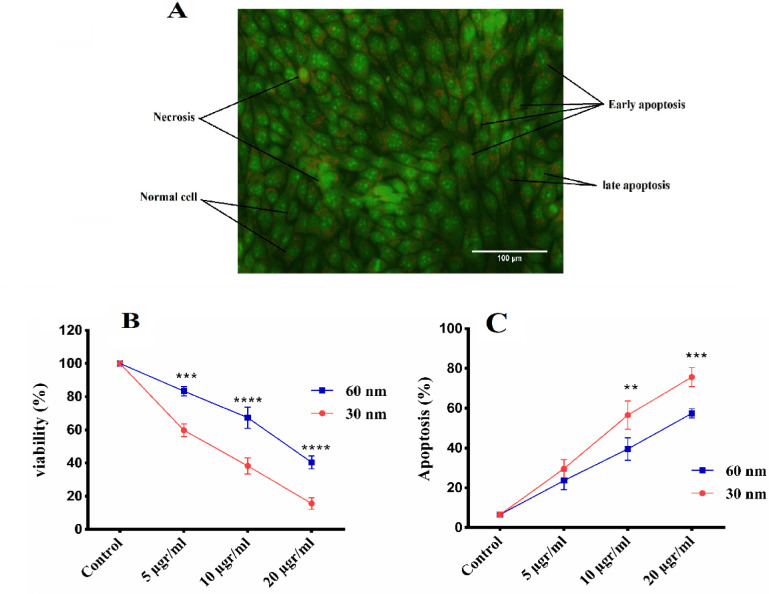
A, apoptosis morphological change of effect different concentration and the different size of CuO NPs on the 4T1 breast cancer with AO/ PI staining by fluorescence microscope. B, Cancer cell viability in exposing different concentrations of CuO NPs in sizes 30 and 60 nm by MTT assay. C, Determination of apoptosis of breast cancer cells treated with different concentrations of CuO NPs in 30 and 60 nm sizes. Data shown are means ± SD (n=3) (*p < 0.05, **p < 0.01, ***p < 0.001 and ****p < 0.0001 vs similar concentrations in different size).

The effect size and concentration of CuO NPs on the viability/proliferation of breast cancer cell line was performed by MTT assay. According to Figure 6 B, increasing the concentration in both sizes has reduced the viability of cancer cells. On the other hand, it has been found that the size of 30 nm has a more significant effect on all concentrations similar to 60 nm of CuO NPs and further reduced the viability/proliferation of breast cancer cells. The half- maximal inhibitory concentration (IC50) indicated at what concentration of CuO NPs inhibited the growth of cancer cells (50%) compare to the untreated cells. The IC_50_ of CuO NPs with size 30 nm were 8.2 ± 2.4, and for CuO NPs with size 60 nm were13.3± 1.8. The IC_50_ has significantly been increased by decreasing particle size. 

*Quantification of cell apoptosis by fluorescence microscopic*

Fluorescence microscopic study was performed to evaluate whether the cytotoxic effect of CuO NPs was related to the stimulation of apoptosis or to the induction of apoptosis. Under the microscope, normal cells were seen as bright green, while early apoptotic cells demonstrated bright green areas of condensed chromatin in the nucleus and necrotic cells represented a uniform bright red nucleus with non-condensed chromatin (include PI). After microscopic examination, Figure 6 C has shown that the rate of apoptosis in 4T1 cancer cells significantly increases in a dose dependent-manner in both CuO NPs concentration. Also, compared with the effect of size of CuO NPs, it has been found that smaller CuO NPs aggregation increases apoptosis in 4T1 cancer cells. Figure 6A shown fluorescence inverts microscopic images of CuO NPs - exposed cells stained by AO/PI.

*Evaluate of malignancy after treatment of 4T1 cell line with CuO NPs*

**Figure 7:**
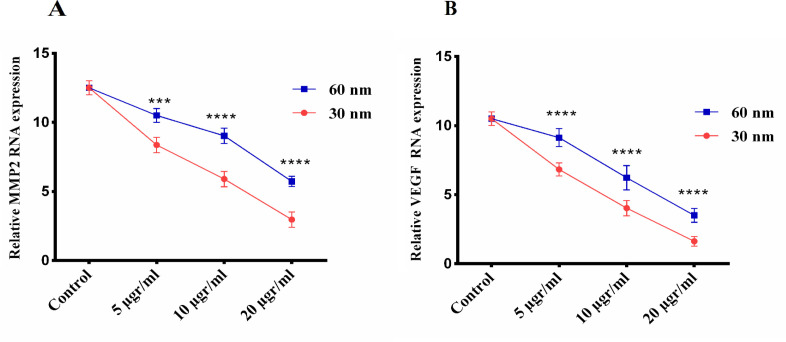
The mRNA Expression Levels of MMP-2 (A) and VEGF (B) in Breast Cancer Cell Treatment with Different Concentrations of CuO NPs in Sizes 30 and 60 nm. HPRT was used for an endogenous control. Data shown are means ± SD (n=3) (*p < 0.05, **p < 0.01, ***p < 0.001 and ****p < 0.0001 vs similar concentrations in different size).

Assessment of malignancy after 4T1 cell line exposed with CuO NPs was based on two indices, including MMP-2 as tumor metastatic potential representative and VEGF as angiogenesis representative for migratory tendency. In Figure 7 A, B demonstrated that in both CuO NPs sizes, increasing the concentration reduced the expression of MMP-2 and VEGF genes in breast cancer cells. Comparing the effect of size on the expression of these genes, it was found that smaller nanoparticles inhibit the expression of genes involved in metastasis and angiogenesis in 4T1 cancer cells. Finally, it was found that CuO NPs with size 30 nm were more effective than CuO NPs with size 60 nm in reducing viability/proliferation, increasing apoptosis, and inhibiting the expression of genes involved in metastasis and angiogenesis in the 4T1 cancer cell line.

**Table 1:** The Results of Disk Diffusion Test and Well Diffusion Tests in the Treatment of *S. epidermidis* and *A. baumannii* Bacteria (Standard and Clinical Strains) with Two Different Sizes of CuO-NPs (30 and 60 nm).

The type of bacteria	Size of Nanoparticle	Disc (mm)	Well Agar (mm)	Negative Control
*S. epidermidis *(Standard)	30 nm	19 ± 1.3	28 ± 1.5	0
	60 nm	14 ± 2	23 ± 2	0
*S. epidermidis* (Clinical)	30 nm	15 ± 1.5	26 ± 2	0
	60 nm	10 ± 2	21 ± 1	0
*A. baumannii* (Standard)	30 nm	13 ± 1	18 ± 1.5	0
	60 nm	9 ± 2.5	14 ± 1.5	0
*A. baumannii *(Clinical)	30 nm	9 ± 1.5	11 ± 2	0
	60 nm	5 ± 2	9 ± 1.5	0

**Table 2:** MIC (μg/ml) and MBC (μg/ml) in the Treatment of *S. epidermidis* and *A. baumannii* Bacteria (Standard and Clinical Strains) with Two Different Sizes of CuO-NPs (30 and 60 nm).

The type of bacteria	Size of Nanoparticle	MIC (g/ml)	MBC (g/ml)
*S. epidermidis* (Standard)	30 nm	2,500	≥5,000
	60 nm	5,000	≥10,000
*S. epidermidis * (Clinical)	30 nm	5,000	≥10,000
	60 nm	10,000	≥10,000
*A. baumannii* (Standard)	30 nm	5,000	≥10,000
	60 nm	10,000	≥10,000
*A. baumannii* (Clinical)	30 nm	10,000	≥10,000
	60 nm	10,000	≥10,000

## Discussion

Despite promising successes in developing new drugs and pharmaceutical biotechnology, infectious diseases and cancer are still the principal causes of mortality and morbidity worldwide. Therefore, finding effective ways to deal with these pathogens and cancers is critical. Metal nanoparticles are one of the new strategies to combat bacteria and cancers. In the present study, investigation on the biological effects of CuO-NPs on bacteria strains, isolated from hospitals, causing nosocomial infections and impacting on cancers, by the antibiogram test, revealed that* S. epidermidis* and *A. baumannii* is resistant to a wide range of antibiotics. Additionally, we have selected breast cancer (4T1) as a growing disease and resistance to available drugs. 

In our study, one-step oxidation of two CuO-NPs with sizes of 30 and 60 nm was made and then evaluated on the antibacterial activities of *S. epidermidis* and *A. baumannii* as an example of gram-positive and negative bacteria responsible for NIs in standard and clinical strains and breast cancer. 

Our results showed that the synthesized nanoparticles were of one size and morphology. Also, in the study of antibacterial effects of disk and well test, it was found that 30 nm nanoparticles had more growth areas than 60 nm nanoparticles with halo diameter. Interestingly, clinical strains illustrated more excellent resistance than standard strains. Based on the results, it could be concluded that the isolates, which are commonplace in the hospital, are developing to be more resistant to antibiotics than the standard strains. It is a warning for the outcome of antibacterial therapeutic strategies. The results also demonstrated that the smaller nanoparticle size had more effects on the resistant strains than the larger nanoparticle size. It has been reported that nanoparticles have some side effects on human cells; hence, using a lower concentration of nanoparticles with more effectiveness is the main goal in antibacterial therapeutic strategies. 

On the other hand, our results demonstrated that similar to antibacterial results, in cytotoxicity tests, apoptosis, and expression of genes responsible for cancer cell metastasis and angiogenesis, in both nanoparticles (30 and 60 nm) with increased concentration led to decreased survival, expression of MMP-2 and VEGF genes and also increased apoptosis in 4T1 cancer cells. Interestingly, 30 nm CuO-NPs at all concentrations compared to 60 nm CuO-NPs have shown a double effect (two folded practical) on anti-cancer and antibacterial properties. Therefore, it appears that lower doses of CuO-NPs in 30 nm can be effective, whereas the 60 nm nanoparticle are beneficial in higher doses. Our results are consistent with those obtained by other researchers, whereas using silver nanoparticles with the size between 20 and 30 nm were associated with an increased inhibitory growth zone on Escherichia coli and S. aureus (Dhand et al., 2016). The beneficial antibacterial effects of gold nanoparticles on pathogenic bacteria have also been reported (Rajan et al., 2015). It has also been shown that 30 nm CuO-NPs are more effective than 60 nm on glioblastoma cancer cells, but there was no significant change (Kukia et al., 2018). These antibacterial and anti-cancer effects of 30 nm in comparison to 60 nm nanoparticles (at smaller sizes compared to larger sizes) could be associated with unique physicochemical properties such as higher surface-to-volume ratios, altered electrical, magnetic, and optical properties and higher reactivity, and greater nanoparticle penetration power (Zhang et al., 2010). Besides, smaller nanoparticles increase the antibacterial and anti-cancer activity of 30 nm by producing cellular ROS, including hydroxyl radical, superoxide anion, and superoxide anion hydrogen peroxide (Belwal et al., 2018). 

Interestingly, in our study, clinical bacterium strains were more resistant than standard strains in both nanoparticle sizes. This is because clinical strains were more exposed to environmental factors than the standard strains and increased pumps on their surface. As well as adaptation to more demanding, conditions resulting in increased resistance to antibiotics and even nanoparticles. On the other hand, it has been shown that the effects of metal nanoparticles on different cancer cell lines differ, which emphasizes the difference in the resistance of cancer cell lines to these nanoparticles.

Collectively, it appears that the 30 nm CuO-NPs nanoparticle can be considered a potential antibacterial agent, especially in the case of resistance to routine antibiotics. Moreover, it can be used to treat metastatic cancers such as breast cancer. However, since nanoparticles can be associated with some complications, it needs to be initially explored in animal models to evaluate possible in vivo side effects. Additionally, this study proposes that 30 nm CuO-NPs could be used to combat metastatic cancer cells, and vehicle disinfectants in hospitals to prevent the growth of resistant bacteria and may include other dangerous biological agents such as viruses (COVID-19) and fungi.

In conclusion, this study suggests that nano-sized nanoparticles (30 or 60 nm) could be synthesized for stability and introduced as an intrinsic and potent antimicrobial and anti-cancer agent. In this study, experiments showed that the 30 nm of CuO-NP had more antibacterial effects against *S. epidermidis* and* A. baumannii* in clinical and standard strains than 60 nm also and anti-cancer effects against breast cancer, possibly due to the increase in size-to-volume ratio and some other physical and chemical properties and even more, is the ability to penetrate the bacterium and cancer cells. Therefore, it can be concluded that CuO-NPs made in a smaller size could be considered an antibacterial and anti-cancer material used in pharmaceutical biotechnology.

## Author Contribution Statement

Study concept and design: Ardeshir Abbasi, and Khoayar Ghorban. Analysis and interpretation of data: Ardeshir Abbasi. Drafting of the manuscript: Ardeshir Abbasi, Khodayar Ghorban, Farshad Nojoomi and Maryam Dadmanesh. Critical revision of the manuscript for important intellectual content: Khodayar Ghorban, Farshad Nojoomi, and Maryam Dadmanesh. 
